# Predicting Laboratory Test Ordering in Emergency Departments Using Integrated Structured and Unstructured Electronic Health Records: Machine Learning Study

**DOI:** 10.2196/85255

**Published:** 2026-06-15

**Authors:** Xingyu Zhang, Haipeng Ling, Xin Zhang, Anao Zhang

**Affiliations:** 1 Department of Communication Science and Disorders, School of Health and Rehabilitation Sciences, University of Pittsburgh, Pittsburgh, PA, United States; 2 Tepper School of Business, Carnegie Mellon University, Pittsburgh, PA, United States; 3 Department of Biostatistics, School of Public Health, University of Michigan, Ann Arbor, MI, United States; 4 Center for Interdisciplinary Psychosocial Oncology and Palliative Care Research and Practice, School of Social Work, University of Washington, 4101 15th Avenue NE, Seattle, WA, 98105, United States, 1 206-543-8867

**Keywords:** clinical decision support, electronic health records, emergency department, laboratory test use, machine learning

## Abstract

**Background:**

Laboratory testing is a cornerstone of diagnostic decision-making in emergency departments (EDs), yet its overuse contributes substantially to unnecessary health care costs and inefficiencies. Predictive approaches that leverage electronic health record data may help optimize and guide more appropriate test use.

**Objective:**

This study aims to develop and evaluate machine learning models that predict laboratory test use during ED visits by integrating structured clinical data and unstructured text from electronic health records.

**Methods:**

We analyzed 13,115 adult ED visits from the 2021 National Hospital Ambulatory Medical Care Survey–Emergency Department dataset. Structured predictors included demographics, vital signs, insurance status, and medical history, while unstructured data from chief complaints and injury descriptions were encoded using Bidirectional Encoder Representations from Transformers–based embeddings. Four model configurations were developed: structured-only, unstructured-only, combined structured and unstructured data, and an ensemble (mean probability) approach. Model performance was evaluated using the area under the receiver operating characteristic curve (AUC).

**Results:**

The combined model achieved the highest predictive performance (AUC=0.83), outperforming both the structured-only model (AUC=0.78) and the unstructured-only model (AUC=0.74). The ensemble model also performed well but did not exceed the combined model. Key predictors of laboratory testing included older age, ambulance arrival, abnormal vital signs, and chronic comorbidities, whereas injury-related visits were associated with a lower likelihood of testing.

**Conclusions:**

Integrating structured and unstructured electronic health record data improves the prediction of laboratory test use in ED settings. These findings support the development of data-driven clinical decision support tools aimed at enhancing diagnostic efficiency and reducing unnecessary testing.

## Introduction

The ordering of laboratory tests is a cornerstone of diagnostic medicine, guiding providers’ clinical decision-making across various healthcare settings, especially in emergency departments (EDs) [1]. In hospitals and EDs, laboratory tests inform the assessment of disease severity, therapeutic decisions, prognosis, and monitoring strategies. However, studies have found that the sheer volume of laboratory orders, including many that may be routine, defensive, or redundant, has raised concerns about overuse, patient burden, and resource inefficiency [2]. This is especially pertinent in EDs, with studies revealing that nearly 80% of patients who present to the ED receive laboratory testing, averaging 7.7 tests per patient [3,4]. Notably, unnecessary laboratory testing contributes to an estimated US $210 billion in additional health care costs annually, underscoring the importance of optimal test use that not only saves costs but also improves ED workflow.

Traditionally, decisions to order laboratory tests are based primarily on clinicians’ judgment, informed by vital signs, presenting symptoms, medical history, and institutional protocols. Yet these decisions can vary significantly across providers and settings, often lacking standardization or transparency [5]. In this context, data-driven approaches—particularly those that leverage electronic health record (EHR) data—offer the potential to identify patterns that precede test ordering, thereby informing real-time decision support tools [6].

Recent developments in machine learning (ML) have enabled predictive modeling of clinical decision points and corresponding actions using *structured data*, such as demographics, vital signs, comorbidity indices, and other variables [7]. Numerous algorithms, such as gradient boosting classifiers, are well-suited for handling heterogeneous tabular data to model the complex interactions between predictors [8]. In addition, a significant portion of providers’ clinical reasoning is captured in *unstructured data*, such as free-text notes, including primary complaints, triage narratives, and clinicians’ nonverbal assessments. Importantly, these unstructured data sources often precede test orders and provide valuable context not captured in structured data fields, underscoring the need and potential to integrate unstructured data into model building [9]. One highly promising approach to achieving this is the use of transformer-based language models.

Although prior studies have explored ML approaches to predict laboratory test ordering, most have relied primarily on structured EHR variables or basic natural language processing (NLP) methods, focusing on single tests or narrow clinical contexts [10,11]. Consequently, the extent to which contextualized information embedded in free-text clinical documentation can meaningfully improve the prediction of general laboratory test use in the ED remains largely underexplored. Addressing this gap is critical to developing scalable, data-driven decision-making tools that better align with real-world clinical reasoning.

Transformer-based language models, such as GPT-2, provide a robust approach for extracting insights from unstructured text sources. By leveraging self-attention mechanisms and deep learning architectures, these models can capture complex linguistic patterns and semantic relationships across large text corpora [12]. Consequently, transformer-based language models can capture the clinical nuances that may influence decisions to order specific laboratory tests, such as concerns about sepsis, renal impairment, or metabolic derangements, among others. Most importantly, ML models that integrate both structured and unstructured data tend to produce the most robust predictive outcomes. With structured data offering a reliable foundation of quantifiable variables, unstructured data captures rich contextual nuances that are often absent in structured formats [13]. As a result, ML algorithms can uncover deeper patterns, improve generalizability, and enhance prediction.

In this study, we aim to develop and evaluate ML models for predicting the likelihood of general laboratory test orders during patients’ ED visits, using both structured EHR data and unstructured clinical text. The models that use structured data alone, unstructured data alone, and a combined approach were evaluated. We hypothesize that integrating structured EHR data with unstructured clinical text will yield more accurate predictions than either source alone, thereby supporting the development of real-time decision tools that can inform laboratory test use in the ED and promote more efficient, patient-centered, and evidence-based care. For this work, the study defines general laboratory tests as a set of commonly ordered panels and individual assays across multiple organ systems and clinical indications, comprising 20 laboratory tests (defined later). By focusing on this representative set, the study aims to capture a wide range of clinical scenarios, including metabolic panels, coagulation profiles, and electrolyte monitoring. Ultimately, this work seeks to improve the prediction of laboratory test use, thereby providing a foundation for further developing and scaling up clinical decision algorithms to support patient care in the ED.

## Methods

### Ethical Considerations

Ethical approval and consent for participation are not applicable, as the research was conducted using publicly available, anonymized data from the NHAMCS-ED (National Hospital Ambulatory Medical Care Survey–Emergency Department) dataset.

### Data Source

We used data from the 2021 NHAMCS-ED dataset [14], a nationally representative dataset that captures a wide range of information about visits to EDs across the United States. NHAMCS-ED is administered by the National Center for Health Statistics and uses a multistage probability sampling design to ensure generalizability to the national population [15]. The dataset includes visit-level records detailing patient demographics, clinical characteristics, reasons for visits, procedures, diagnostic tests, medications, and disposition. For this study, the sample was restricted to adult patients aged 18 years or older. The study excluded visits with missing data on our primary outcome of interest—laboratory test use—as well as records with critical missing values for key predictor variables. The final analytic cohort comprised all eligible adult ED visits for which complete information on laboratory testing and associated predictors was available.

### Outcome Variable: Laboratory Test Use

Laboratory test use was operationalized as a binary outcome, indicating whether one or more laboratory tests from a predefined set of commonly ordered assays were prescribed during an ED visit. Using available NHAMCS-ED variables and informed by prior literature, we identified 20 laboratory tests and panels spanning multiple organ systems and clinical indications, such as metabolic, hematologic, renal, hepatic, and coagulation-related tests.

For each visit, test ordering was coded as “1” if at least one of these tests or panels was ordered and “0” otherwise. Visits involving multiple tests or panels were treated as positive events without additional weighting, as the objective was to capture the occurrence of laboratory evaluation rather than test volume, sequencing, or clinical appropriateness. Because there is no widely accepted approach for encoding the complex combinations and ordering patterns of laboratory tests in large-scale ED datasets, this binary operationalization is a pragmatic, scalable strategy that avoids arbitrary weighting while supporting robust predictive modeling.

### Structured Predictors

Structured predictors were drawn from multiple domains within the NHAMCS dataset. These included demographic variables (eg, age, sex, race, or ethnicity), visit characteristics (eg, day of the week, time of arrival, arrival by ambulance, and mode of arrival), and vital signs (eg, heart rate, blood pressure, respiratory rate, temperature, oxygen saturation, and pain level).

We also included a comprehensive set of medical history variables, coded as binary indicators for the presence of specific chronic or acute conditions. These included Alzheimer disease or dementia, asthma, cancer, cerebrovascular disease, chronic kidney disease, chronic obstructive pulmonary disease, congestive heart failure, coronary artery disease, diabetes mellitus (types I and II), end-stage renal disease, HIV/AIDS, hyperlipidemia, hypertension, obesity, obstructive sleep apnea, osteoporosis, depression, substance abuse or dependence, and thromboembolic conditions such as deep vein thrombosis or pulmonary embolism.

Additional structured features included insurance type (private, Medicare, Medicaid, Children's Health Insurance Program, uninsured, or other); residence type (private home, nursing home, homeless, or other); whether the visit was a follow-up or related to an earlier episode of care; and whether the visit involved injury or trauma, poisoning or overdose, or adverse effects of treatment. These structured features were selected based on prior literature, clinical rationale, and exploratory data analysis.

Prior to modeling, continuous variables were standardized using the StandardScaler method to facilitate model convergence and ensure comparable feature-importance estimates across models.

Missing data analysis revealed that, except for the pain score variable (with 38.3% missing), all structured features exhibited low to moderately low levels of missingness, that is, less than 10% (Multimedia Appendix 1). To assess the plausibility of the missing-at-random assumption, we examined patterns of missingness in relation to laboratory test use and observable visit characteristics for the pain score variable. Notably, the missingness of the pain score variable was not associated with laboratory testing rates (ie, 59.6% for observed vs 59% for missing), and was more frequent among ambulance arrivals, consistent with higher-acuity encounters in which clinical priorities may precede formal pain documentation.

For other administrative and visit-level variables, differences in laboratory testing rates between missing and observed groups were very low to modest. Larger differences were observed for vital signs, such as temperature, oxygen saturation, and pulse, reflecting ED workflows in which these parameters are preferentially measured in higher-acuity encounters, which are also more likely to undergo laboratory evaluation. Taken together, these patterns suggest that missingness in structured variables is largely driven by observable clinical context and documentation practices rather than outcome-dependent mechanisms, thereby supporting the use of median imputation under the missing-at-random assumption.

### Unstructured Data and Bidirectional Encoder Representations from Transformers Model

The NHAMCS-ED dataset includes two primary unstructured text fields: (1) the chief complaint, which captures the patient’s primary reason for visiting the ED, and (2) the reason-for-visit injury text, which describes any injury-related circumstances. These free-text fields provide rich clinical context often unavailable in structured variables and are frequently used by ED staff to understand the patient’s presenting condition.

To process these fields, we applied a text-cleaning pipeline to enhance consistency and remove irrelevant noise [16]. This included converting all text to lowercase, removing punctuation and numerals, stripping whitespace, and filtering out common stopwords (eg, “and,” “the,” “of”) using a predefined list from the Natural Language Toolkit [17]. The resulting cleaned text provided the input for our NLP pipeline.

We used a pretrained Bidirectional Encoder Representations from Transformers (BERT) model from the HuggingFace *Transformers* library to encode the cleaned text [18]. BERT is a state-of-the-art transformer-based language model that learns deep contextual relationships between words through bidirectional attention. The BERT-base-uncased model and its tokenizer were used to convert each text entry into input tokens compatible with the BERT model architecture [19].

These tokenized inputs were then passed through BERT to extract sentence-level embeddings—dense vector representations capturing the semantic content of the chief complaint and injury text. Performance was robust to alternative text-embedding pooling ([CLS], also known as classification, token vs mean pooling). These embeddings, typically 768-dimensional vectors, served as the unstructured input features for downstream modeling. They enabled us to leverage the rich semantic meaning encoded in patient narratives, augmenting the structured clinical features.

To assess whether our reliance on the [CLS] embedding affected model performance, we also evaluated an alternative, widely used aggregation strategy: mean pooling over token embeddings [20]. The 2 approaches yielded highly comparable results in the gradient boosting classifier (receiver operating characteristic curve, AUC=0.83 for [CLS] vs 0.84 for mean pooling), indicating that sentence-level performance was robust to the choice of pooling method. Given the comparison result, we retained the [CLS] representation as our primary specification, consistent with standard BERT practice for sequence-level encoding [21].

### Association Analysis

To identify factors independently associated with laboratory test use, a logistic regression with Lasso regularization was performed for variable selection [22]. This approach penalizes less informative predictors and retains only those with the strongest associations, helping reduce overfitting and improving model interpretability. After selecting variables with nonzero coefficients from the Lasso model, a standard multivariable logistic regression was fitted using the selected predictors to estimate adjusted odds ratios (ORs) and 95% CIs. A forest plot was generated to visualize the direction and strength of associations. Variables with ORs above 1 were associated with higher odds of laboratory testing, while ORs below 1 indicated a reduced likelihood. This analysis highlighted key clinical and demographic drivers of test-ordering decisions in the ED.

### Predictive Model Development

To predict laboratory test use during ED visits, we developed 4 supervised ML models: logistic regression, random forest, gradient boosting, and extreme gradient boosting. These models were selected to capture both linear and complex nonlinear relationships among predictors. Logistic regression served as a benchmark model due to its interpretability, while the ensemble-based models (random forest, gradient boosting, and extreme gradient boosting) offered greater flexibility in capturing variable interactions and improving classification performance.

Each model was applied to four configurations of input data: (1) structured data only, including demographics, vitals, medical history, and visit details; (2) unstructured text data only, using BERT-generated embeddings from chief complaints and reasons for visit; (3) combined structured and unstructured data; and (4) a mean probability model, which averaged predicted probabilities from the structured-only and unstructured-only models.

Structured data were preprocessed using median imputation and standardized for modeling. Unstructured data were cleaned, tokenized, and processed through a pretrained BERT model to generate contextual embeddings. All models were trained and evaluated using 5-fold cross-validation to ensure robust performance estimates across data splits.

### Evaluation Metrics

Model performance was evaluated using standard classification metrics: area under the receiver operating characteristic (ROC) curve (AUC) [23], accuracy, precision, recall (sensitivity), specificity, and *F*_1_-score [24]. AUC was the primary measure of discrimination, reflecting the model’s ability to distinguish between visits with and without laboratory testing. Precision and recall offered insights into the model’s accuracy in predicting test use, with the *F*_1_-score providing a balanced measure of both.

We also examined specificity to assess how well the models avoided false positives—cases where testing was not conducted but was predicted as such. Optimal classification thresholds were determined using the point on the ROC curve closest to the top-left corner.

To aid interpretation, we visualized logistic regression coefficients using forest plots and generated word clouds from unstructured data to highlight common terms associated with laboratory testing decisions.

### Model Interpretability

As an important extension of our analysis, feature contributions were quantified using Shapley additive explanations (SHAP) [25]. For tree-based models, TreeExplainer was used and for logistic regression [26], the linear explainer was used with the model’s logit link [27]. To synthesize the unstructured input (ie, BERT embeddings), we computed SHAP values and aggregated them into a single textual feature by summing the mean absolute SHAP values across all dimensions, reported as Combined_Chief_Complaint.

## Results

### Descriptive Statistics and Feature Selection

Among adult ED visits in the 2021 NHAMCS-ED dataset, 7784 (59.3%) visits included laboratory test orders. Significant differences were observed across nearly all demographic and clinical variables between patients who received laboratory tests and those who did not (Table 1). Patients who underwent laboratory testing were more likely to be female (4341/7784, 55.8% vs 2741/5331, 51.4%) and older, with 2298 out of 7784 (29.5%) aged 65 years or older compared to 834 out of 5331 (15.6%) in the no-test group. The proportion of White patients was slightly higher in the tested group (4779/7784, 61.4% vs 2958/5331, 55.5%), while Black and Hispanic patients were slightly underrepresented. Laboratory-tested patients were more likely to reside in nursing homes (208/7784, 2.7% vs 58/5331, 1.1%) and to be insured through Medicare (2296/7784, 29.5% vs 905/5331, 16.9%), while the no-test group had higher proportions of Medicaid (1766/5331, 33.1% vs 2033/7784, 26.1%) and uninsured individuals (480/5331, 9% vs 461/7784, 5.9%). Patients with laboratory testing were more frequently transported by ambulance (2012/7784, 25.8% vs 682/5331, 12.7%) and less likely to have injury-related visits (1015/7784, 13% vs 2137/5331, 40%). Clinical severity indicators also differed: higher proportions of laboratory-tested patients had abnormal vital signs, including heart rates of more than 90 bpm (3110/7784, 39.9% vs 1712/5331, 32.1%), diastolic blood pressure of less than 60 mm Hg (536/7784, 6.9% vs 218/5331, 4.1%), oxygen saturation of less than 95% (996/7784, 12.8% vs 297/5331, 5.6%), and respiratory rates of more than 20 breaths/minute (914/7784, 11.7% vs 210/5331, 3.9%). Patients who received laboratory tests had a higher prevalence of chronic conditions, including hypertension (3116/7784, 40% vs 1183/5331, 22.2%), diabetes (type II: 936/7784, 12% vs 301/5331, 5.6%), coronary artery disease (839/7784, 10.8% vs 192/5331, 3.6%), chronic kidney disease (537/7784, 6.9% vs 100/5331, 1.9%), congestive heart failure (569/7784, 7.3% vs 110/5331, 2.1%), and chronic obstructive pulmonary disease (684/7784, 8.8% vs 231/5331, 4.3%). Other conditions significantly more common in the laboratory-tested group included cancer, dementia, stroke, end-stage renal disease, and hyperlipidemia.

**Table 1.** Demographic and clinical profiles of emergency department patients stratified by laboratory test use (N=13,115)^a^.

	No laboratory test (n=5331), n (%)	Laboratory test (n=7784), n (%)	*P* value
Gender			<.001
Female	2741 (51.4)	4341 (55.8)	
Male	2590 (48.6)	3443 (44.2)	
Age (y)			<.001
18‐39	2606 (48.9)	2553 (32.8)	
40‐65	1891 (35.5)	2933 (37.7)	
≥65	834 (15.6)	2298 (29.5)	
Race or ethnicity			<.001
White	2958 (55.5)	4779 (61.4)	
Black	1348 (25.3)	1675 (21.5)	
Hispanic	804 (15.1)	1023 (13.1)	
Other	221 (4.1)	307 (3.9)	
Residence type			<.001
Private residence	4879 (94.5)	7173 (94.2)	
Nursing home	58 (1.1)	208 (2.7)	
Homeless	169 (3.3)	145 (1.9)	
Other	56 (1.1)	88 (1.2)	
Insurance type			<.001
Private insurance	1385 (29)	2097 (29.6)	
Medicare	905 (16.9)	2296 (29.5)	
Medicaid or CHIP^b^	1766 (33.1)	2033 (26.1)	
Uninsured	480 (9)	461 (5.9)	
Other	243 (5.1)	207 (2.9)	
Day of week			.07
Weekdays	3937 (75.8)	5873 (77.2)	
Weekend	1256 (24.2)	1734 (22.8)	
Arrival time			.32
Morning	1459 (28.3)	2228 (29.2)	
Afternoon	1584 (30.7)	2396 (31.4)	
Evening	839 (16.3)	1173 (15.4)	
Night	1278 (24.8)	1840 (24.1)	
Arrive by ambulance	682 (12.7)	2012 (25.8)	<.001
Follow-up visit	422 (8.6)	490 (6.9)	.009
Seen within last 72 hours	201 (4.2)	301 (4.2)	>.99
Pain level			<.001
No or mild pain	1159 (35.4)	1965 (40.8)	
Moderate or severe pain	1090 (33.3)	1521 (31.6)	
Very severe and unbearable pain	1024 (31.3)	1333 (27.7)	
Temperature (°C)			<.001
36‐38	4710 (95.9)	6920 (93.9)	
≤36	149 (3)	266 (3.6)	
>38	51 (1)	186 (2.5)	
Heart rate, beats per minute			<.001
≤60	204 (4.2)	358 (4.8)	
61‐90	2983 (60.9)	4051 (53.9)	
>90	1712 (32.1)	3110 (39.9)	
DBP^c^ (mm Hg)			<.001
<60	218 (4.1)	536 (6.9)	
60‐80	2192 (44.1)	3373 (44.4)	
>80	2556 (51.5)	3680 (48.5)	
SBP^d^ (mm Hg)			<.001
<80	1142 (23)	1761 (23.2)	
80‐120	1 (0)	23 (0.3)	
>120	3827 (77)	5805 (76.5)	
Pulse oximetry (%)			<.001
0‐94	297 (5.6)	996 (12.8)	
≥95	4549 (93.9)	6496 (86.7)	
Respiratory rate per minute			<.001
<12	16 (0.3)	27 (0.4)	
12‐20	4695 (95.4)	6588 (87.5)	
>20	210 (3.9)	914 (11.7)	
Injury status			<.001
Yes, injury or trauma	2137 (40)	1015 (13)	
Yes, overdose or poisoning	56 (1.1)	83 (1.1)	
Yes, adverse effect of medical or surgical treatment	125 (2.5)	236 (3.2)	
No	2593 (51.7)	5948 (79.9)	
Questionable injury status	106 (2.1)	165 (2.2)	
Medical history			
Alzheimer disease or dementia	44 (0.8)	162 (2.1)	<.001
Asthma	553 (10.4)	849 (10.9)	.35
Cancer	138 (2.6)	520 (6.7)	<.001
Cerebrovascular disease or history of stroke (CVA^e^)	125 (2.3)	457 (5.9)	<.001
CKD^f^	100 (1.9)	537 (6.9)	<.001
COPD^g^	231 (4.3)	684 (8.8)	<.001
CHF^h^	110 (2.1)	569 (7.3)	<.001
CAD^i^	192 (3.6)	839 (10.8)	<.001
Depression	664 (12.5)	1306 (16.8)	<.001
Diabetes mellitus type I	25 (0.5)	77 (1)	<.001
Diabetes mellitus type II	301 (5.6)	936 (12)	<.001
ESRD^j^	25 (0.5)	160 (2.1)	<.001
PE^k^, DVT^l^, or VTE^m^	74 (1.4)	207 (2.7)	<.001
HIV infection/AIDS	58 (1.1)	83 (1.1)	.97
Hyperlipidemia	413 (7.7)	1339 (17.2)	<.001
Hypertension	1183 (22.2)	3116 (40)	<.001
Obesity (BMI ≥30)	328 (6.2)	775 (10)	<.001
OSA^n^	113 (2.1)	341 (4.4)	<.001
Osteoporosis	48 (0.9)	111 (1.4)	.009
Substance abuse or dependence	517 (9.7)	795 (10.2)	.35

a The variables “Respiratory rate,” “Temperature,” “Pulse oximetry,” “Heart rate,” “Payment type,” “Seen within last 72 hours,” and “Episode of care” have missing data proportions ranging between 5% and 10%. The variables “Arrival time,” “Patient residence,” “Arrival by ambulance,” “Systolic blood pressure,” “Diastolic blood pressure,” and “Visit related to injury or trauma, overdose or poisoning, or adverse effect of medical or surgical treatment” have missing data proportions of less than 5%.

b CHIP: Children’s Health Insurance Program.

c DBP: diastolic blood pressure.

d SBP: systolic blood pressure.

e CVA: cerebrovascular accident.

f CKD: chronic kidney disease.

g COPD: chronic obstructive pulmonary disease.

h CHF: congestive heart failure.

i CAD: coronary artery disease.

j ESRD: end-stage renal disease.

k PE: pulmonary embolism.

l DVT: deep vein thrombosis.

m VTE: venous thromboembolism.

n OSA: obstructive sleep apnea.

**Figure 1.**
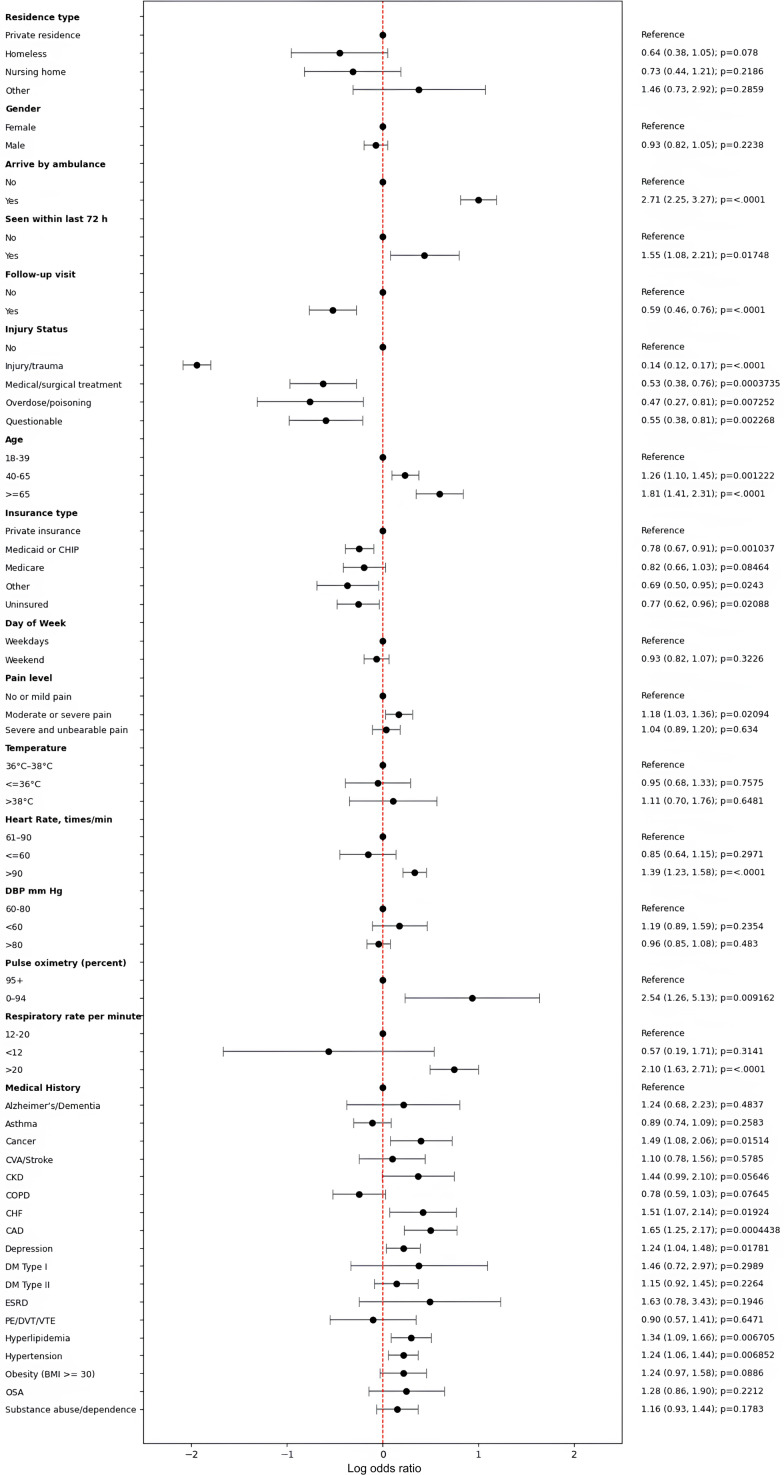
Forest plot of odds ratios with 95% CI (log scale). CKD: chronic kidney disease; CHF: congestive heart failure; COPD: chronic obstructive pulmonary disease; CVA: cerebrovascular disease or history of stroke; CAD: coronary artery disease; DM: diabetes mellitus; ESRD: end-stage renal disease; OSA: obstructive sleep apnea; PE: pulmonary embolism; VTE: venous thromboembolism.

Figure 1 displays adjusted odds ratios (ORs) and 95% CIs for predictors of laboratory test use during ED visits, derived from Lasso-selected logistic regression models. Older age, particularly aged 65 years and older (OR 2.30, 95% CI 2.01‐2.62), and arrival by ambulance (OR 2.31, 95% CI 2.07‐2.58) were among the strongest predictors of laboratory test use. Patients with chronic conditions such as hypertension (OR 1.87, 95% CI 1.68‐2.08), type II diabetes (OR 1.69, 95% CI 1.47‐1.93), chronic kidney disease (OR 2.12, 95% CI 1.75‐2.56), and cancer (OR 1.85, 95% CI 1.48‐2.30) had significantly higher odds of receiving laboratory tests. In contrast, visits related to injury or trauma were associated with substantially lower odds of testing (OR 0.39, 95% CI 0.35‐0.43). Elevated heart rate (>90 bpm) (OR 1.32, 95% CI 1.20‐1.45), low diastolic blood pressure (<60 mm Hg) (OR 1.36, 95% CI 1.15‐1.60), respiratory rate of more than 20 breaths/minute (OR 1.66, 95% CI 1.37‐2.02), temperature of more than 38°C (OR 1.87, 95% CI 1.32‐2.65), and oxygen saturation of less than 95% (OR 2.38, 95% CI 2.03‐2.78) were all significantly associated with increased odds of laboratory testing, reflecting the influence of clinical instability on diagnostic intensity.

**Figure 2.**
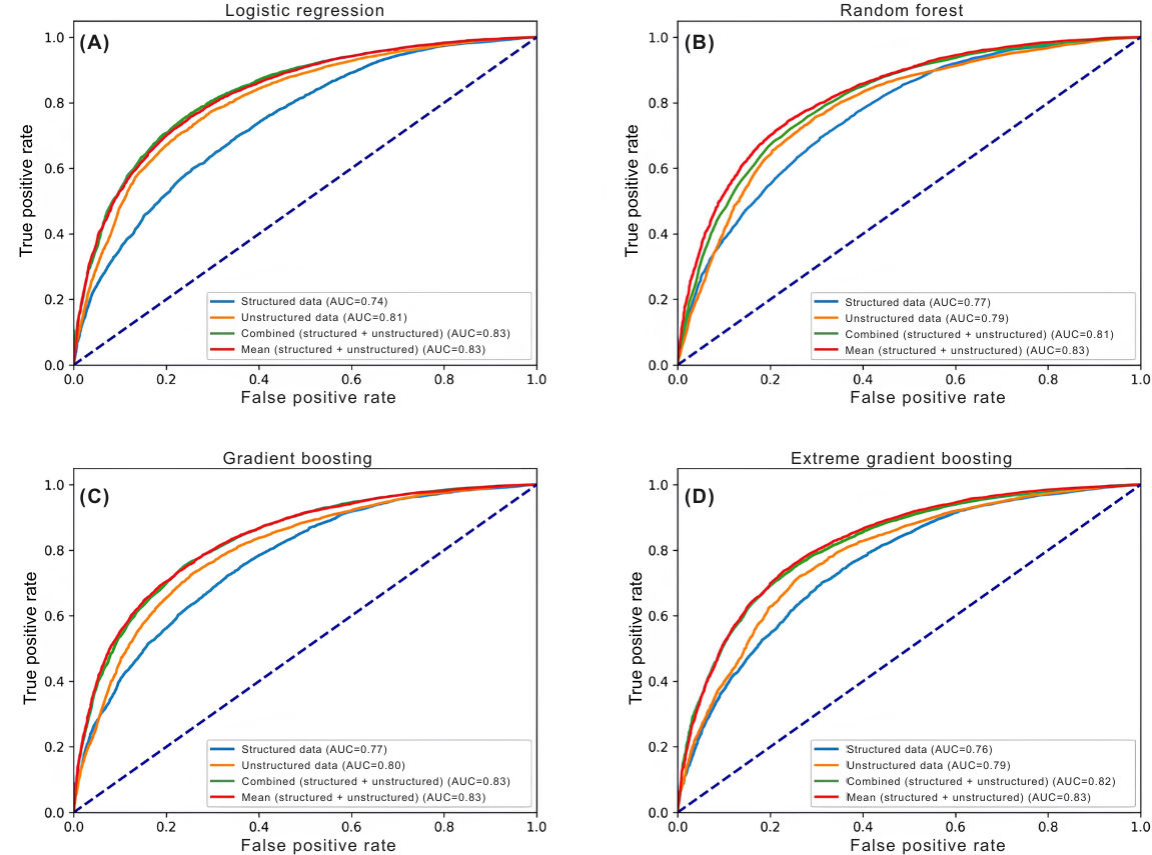
Receiver operating characteristic (ROC) curves for the 4 models evaluated in the study. Each model includes the structured data model, which uses only structured data such as patient demographics, visit characteristics, vital signs, and medical history; the unstructured data model, a Bidirectional Encoder Representations from Transformers (BERT)–based natural language processing (NLP) model that uses only unstructured data, including chief complaints and reasons for injury; the combined input model, a machine learning (ML) classification model that integrates both structured data and BERT-extracted features from the unstructured data; and the mean probability model, which averages the predicted probabilities from the structured data model and the unstructured data model. (A) Logistic regression, (B) random forest, (C) gradient boosting, and (D) extreme gradient boosting. AUC: area under the ROC curve.

Figure 2 illustrates the ROC curves for the 4 ML models developed to predict laboratory test use during ED visits. These models included (1) a structured-only model, which used demographic, clinical, and visit-level features; (2) an unstructured-only model, which used BERT-derived embeddings from free-text chief complaints and reason-for-visit narratives; (3) a combined model, which integrated both structured and unstructured inputs; and (4) a mean probability model, which averaged the predicted probabilities from the structured-only and unstructured-only models without retraining.

Among the models, the combined model demonstrated the highest predictive performance, with an AUC of 0.83, indicating excellent discrimination between visits with and without laboratory test orders. This performance reflects the model’s ability to capture both quantifiable patient characteristics and nuanced clinical context embedded in free-text narratives. The structured-only model achieved a slightly lower AUC of 0.78, suggesting that structured data alone provided a strong baseline for prediction but lacked the richness conveyed in narrative data. The unstructured-only model, based entirely on transformer-derived text embeddings, yielded an AUC of 0.74. While lower than the structured and combined models, it still demonstrated that free-text chief complaints and injury descriptions carry meaningful predictive signals for test use, especially in the absence of structured data. The mean probability model—a late-fusion ensemble averaging outputs from the structured-only and unstructured-only models—achieved an AUC of 0.81, performing better than either source alone but slightly below the fully integrated combined model. This result underscores the benefit of model ensembling, even without retraining or direct feature integration. All detailed performance metrics are presented in Table 2.

**Table 2.** All model performance metrics and their corresponding 95% CI.

Model	AUC^a^	Accuracy	Precision	Recall (sensitivity)	Specificity	*F*_1_-score	Cutoff
Gradient boosting							
Structured data	0.764 (0.755‐0.772)	0.687 (0.681‐0.701)	0.764 (0.748‐0.772)	0.684 (0.676‐0.728)	0.691 (0.650‐0.701)	0.721 (0.715‐0.743)	0.601 (0.581‐0.604)
Unstructured data	0.801 (0.793‐0.808)	0.736 (0.728‐0.743)	0.804 (0.795‐0.815)	0.734 (0.719‐0.748)	0.739 (0.724‐0.755)	0.767 (0.758‐0.775)	0.599 (0.587‐0.608)
Combined (structured + unstructured)	0.830 (0.823‐0.837)	0.754 (0.746‐0.762)	0.817 (0.807‐0.827)	0.754 (0.737‐0.778)	0.754 (0.732‐0.772)	0.784 (0.775‐0.794)	0.600 (0.575‐0.612)
Mean (structured + unstructured)	0.830 (0.823‐0.837)	0.750 (0.744‐0.760)	0.824 (0.806‐0.831)	0.735 (0.728‐0.773)	0.771 (0.735‐0.781)	0.777 (0.771‐0.792)	0.616 (0.592‐0.621)
Extreme boosting							
Structured data	0.745 (0.735‐0.753)	0.679 (0.670‐0.688)	0.746 (0.736‐0.756)	0.697 (0.681‐0.710)	0.654 (0.641‐0.671)	0.721 (0.710‐0.729)	0.598 (0.591‐0.609)
Unstructured data	0.786 (0.777‐0.794)	0.726 (0.718‐0.735)	0.798 (0.788‐0.807)	0.722 (0.708‐0.738)	0.733 (0.716‐0.747)	0.758 (0.749‐0.766)	0.653 (0.628‐0.684)
Combined (structured + unstructured)	0.821 (0.813‐0.828)	0.749 (0.741‐0.757)	0.814 (0.803‐0.823)	0.747 (0.733‐0.771)	0.751 (0.728‐0.767)	0.779 (0.771‐0.790)	0.652 (0.607‐0.678)
Mean (structured + unstructured)	0.818 (0.811‐0.825)	0.746 (0.739‐0.756)	0.810 (0.798‐0.818)	0.748 (0.738‐0.773)	0.744 (0.720‐0.756)	0.778 (0.770‐0.788)	0.613 (0.594‐0.621)
Random forest							
Structured data	0.762 (0.754‐0.771)	0.688 (0.681‐0.704)	0.762 (0.746‐0.773)	0.691 (0.676‐0.736)	0.684 (0.643‐0.702)	0.725 (0.716‐0.747)	0.600 (0.582‐0.606)
Unstructured data	0.788 (0.780‐0.796)	0.727 (0.721‐0.740)	0.799 (0.783‐0.807)	0.722 (0.715‐0.761)	0.735 (0.697‐0.746)	0.759 (0.752‐0.777)	0.594 (0.567‐0.595)
Combined (structured + unstructured)	0.812 (0.804‐0.819)	0.737 (0.730‐0.748)	0.807 (0.793‐0.818)	0.732 (0.715‐0.763)	0.744 (0.714‐0.765)	0.768 (0.758‐0.781)	0.593 (0.580‐0.610)
Mean (structured + unstructured)	0.825 (0.817‐0.832)	0.748 (0.741‐0.758)	0.824 (0.808‐0.835)	0.732 (0.718‐0.765)	0.772 (0.738‐0.790)	0.775 (0.767‐0.788)	0.610 (0.588‐0.616)
Logistic regression							
Structured data	0.743 (0.734‐0.752)	0.674 (0.662‐0.685)	0.744 (0.734‐0.761)	0.687 (0.636‐0.714)	0.655 (0.633‐0.706)	0.714 (0.692‐0.728)	0.586 (0.575‐0.610)
Unstructured data	0.807 (0.799‐0.814)	0.741 (0.730‐0.749)	0.801 (0.792‐0.819)	0.749 (0.706‐0.770)	0.729 (0.710‐0.771)	0.774 (0.757‐0.784)	0.589 (0.570‐0.641)
Combined (structured + unstructured)	0.831 (0.824‐0.838)	0.760 (0.750‐0.768)	0.815 (0.806‐0.832)	0.771 (0.733‐0.784)	0.745 (0.730‐0.780)	0.792 (0.778‐0.800)	0.584 (0.569‐0.635)
Mean (structured + unstructured)	0.828 (0.820‐0.834)	0.749 (0.742‐0.761)	0.822 (0.804‐0.831)	0.735 (0.722‐0.775)	0.768 (0.730‐0.782)	0.776 (0.769‐0.793)	0.614 (0.585‐0.625)

a AUC: area under the receiver operating characteristic curve.

### Global Feature Importance

SHAP analysis findings were consistent with logistic regression. The Combined_Chief_Complaint was the most influential feature across all models. Similar findings were observed across models; the most influential features included age, a visit related to injury, poisoning, or an adverse effect of medical or surgical treatment, ambulance arrival, and vital signs (hypertension, chronic kidney disease, and diabetes). Table 3 presents all SHAP values across models.

**Table 3.** Shapley additive explanations (SHAP) values across machine learning (ML) models^a^^,^^b^

ML model and feature group	Mean |SHAP|
Gradient boosting	
CCC^c^	2.4591
Age (y)	0.2100
ABA^d^	0.1736
VRIPA^e^	0.1734
Hypertension	0.1211
Heart rate	0.1100
Respiratory rate	0.0399
Insurance type	0.0393
Pulse oximetry (%)	0.0379
Diastolic blood pressure	0.0262
Hyperlipidemia	0.0222
Body temperature	0.0206
Coronary artery disease	0.0206
Month of ED^f^ visit	0.0202
Type I diabetes	0.0122
Chronic kidney disease	0.0121
Congestive heart failure	0.0110
Systolic blood pressure	0.0110
Cancer	0.0093
Type II diabetes	0.0087
Extreme boosting	
CCC	9.7059
Age (y)	0.3059
ABA	0.2658
VRIPA	0.2445
Heart rate	0.1921
Hypertension	0.1593
Systolic blood pressure	0.1228
Pulse oximetry (%)	0.1146
Body temperature	0.1117
Diastolic blood pressure	0.0987
Insurance type	0.0943
Respiratory rate	0.0932
Month of ED visit	0.0903
Race or ethnicity	0.0675
Coronary artery disease	0.0587
Pain level at triage	0.0392
Type II diabetes	0.0367
Type I diabetes	0.0360
Hyperlipidemia	0.0357
Day of week of visit	0.0343
Random forest	
CCC	0.2726
VRIPA	0.0525
Age (y)	0.0427
ABA	0.0281
Hypertension	0.0275
Heart rate	0.0178
Insurance type	0.0142
Respiratory rate	0.0118
Pulse oximetry (%)	0.0116
Month of ED^f^ visit	0.0094
Hyperlipidemia	0.0067
Race or ethnicity	0.0060
Sex	0.0036
Coronary artery disease	0.0034
Type II diabetes	0.0029
EOC^g^	0.0021
Congestive heart failure	0.0019
Depression	0.0019
Chronic kidney disease	0.0018
Type I diabetes	0.0016
Logistic regression	
CCC	43.6904
Age (y)	0.2433
ABA	0.2333
VRIPA	0.2011
Heart rate	0.1558
Hypertension	0.1146
Month of ED visit	0.0751
Type 1 diabetes	0.0616
EOC	0.0547
Hyperlipidemia	0.0509
Race or ethnicity	0.0509
Diastolic blood pressure	0.0464
Cancer	0.0423
Body temperature	0.0423
Pain level at triage	0.0417
Depression	0.0396
Insurance type	0.0392
Chronic kidney disease	0.0378
Congestive heart failure	0.0374
Coronary artery disease	0.0358

a SHAP values for textual input were aggregated by summing the mean absolute SHAP values across all BERT embedding dimensions, treating the text as a single composite feature, that is, CCC, VRIPA, ABA, and EOC.

b The reference group is “2016-only (no revisits in 2017–2019)”.

c CCC: Combined_Chief_Complaint.

d ABA: arrival by ambulance.

e VRIPA: visit related to injury, poisoning.

f ED: emergency department.

g EOC: Episode of Care.

## Discussion

### Summary of Main Findings

This study developed and evaluated ML models to predict whether general laboratory tests are ordered during ED visits and found that combining structured EHR variables with unstructured clinical text yields the strongest predictive accuracy. Across model families, the combined approach consistently outperformed models built on structured-only or unstructured-only inputs, underscoring the complementary value of integrating quantifiable patient factors with the contextual nuance present in free-text narratives. Notably, such a finding builds on and further affirms the existing methodological literature suggesting the potential of combining structured and unstructured data [28,29].

Methodologically, our work extends prior studies that either relied exclusively on structured EHR data or incorporated unstructured text using basic NLP techniques. By leveraging contextualized language model embeddings (BERT) alongside structured features and comparing structured-only, unstructured-only, and combined inputs within a common framework, we demonstrate the added value of free-text clinical narratives for laboratory-order prediction at scale. In 5-fold cross-validation, the combined model achieved an AUC of 0.83, compared with 0.78 for structured-only, 0.74 for unstructured-only, and 0.81 for a simple late-fusion mean-probability ensemble, indicating that feature-level integration captures informative cross-modal interactions beyond the ensemble alone. Performance was robust to alternative text-embedding pooling strategies ([CLS] vs mean pooling), suggesting that gains are not an artifact of a particular representation choice. Model interpretability analyses were concordant across methods: the aggregated text-embedding feature and clinically intuitive predictors (eg, age, ambulance arrival, injury indicator, comorbidities, and vital signs) were among the most influential contributors, consistent with associations observed in Lasso-selected logistic regression.

Across all ML model families, SHAP analyses demonstrated a highly consistent set of influential predictors, including patient age, arrival by ambulance, abnormal vital signs (notably tachycardia, tachypnea, hypoxia, and fever), chronic comorbidity burden, and injury-related visit status. These findings align closely with established emergency medicine literature showing that older age, higher acuity of arrival, and physiologic instability are central drivers of diagnostic intensity and laboratory use in ED populations [30,31]. Large observational studies and multicenter cohort analyses have consistently demonstrated that derangements in initial vital signs are strongly associated with downstream diagnostic evaluation, admission, and short-term adverse outcomes, particularly among older and medically complex patients [32]. Similarly, chronic conditions such as diabetes, hypertension, and chronic kidney disease have been shown to increase clinicians’ diagnostic vigilance due to higher baseline risk, atypical presentations, and concern for occult metabolic or infectious pathology, reinforcing their consistent importance across predictive models [33].

In contrast, injury-related or trauma-related visits were consistently associated with a lower predicted likelihood of laboratory testing, reflecting well-described trauma workflows that emphasize imaging, procedural assessment, and protocolized resuscitation over broad laboratory evaluation in the absence of physiologic instability [34,35]. Prior trauma-focused studies have shown that routine laboratory testing in low-to-moderate-severity trauma often yields limited actionable information, supporting more selective testing strategies [36]. Notably, the aggregated unstructured text feature (Combined Chief Complaint) emerged as the most influential predictor across all models, underscoring the critical role of free-text documentation in capturing early diagnostic reasoning. Recent explainable machine-learning studies in emergency care have demonstrated that clinical narratives encode nuanced contextual signals—such as concern for infection, metabolic disturbance, or cardiorespiratory compromise—that may precede or extend beyond structured data in shaping test-ordering behavior [28,33]. Together, these findings indicate that integrating unstructured clinical text not only improves predictive performance but also closely mirrors real-world clinical reasoning during early ED decision-making.

### Clinical Implications

These findings have practical implications for diagnostic stewardship in ED settings. Because unnecessary or default laboratory testing contributes to avoidable costs, patient burden, and workflow inefficiency, predictive models that identify encounters with a lower likelihood of testing could serve as a lightweight prompt for clinicians to reassess routine or defensive ordering. In this way, model outputs can support more efficient, evidence-informed, and patient-centered care while preserving clinician judgment [37].

The results also highlight the use of transformer-based language models in clinical prediction tasks: contextual embeddings capture linguistic complexity and semantic relationships in chief complaints and triage narratives, such as early concern for infection, hemodynamic instability, or metabolic disturbance, which may precede and inform ordering decisions in ways not fully represented by structured data [38]. As large language models trained on clinical corpora continue to evolve, there is potential to further improve performance, interpretability, and operational usability for diagnostic stewardship applications.

Beyond model performance, we observed differences in laboratory testing by race and insurance status that mirror longstanding variations in diagnostic intensity across US EDs. Prior work has documented that minoritized racial groups often receive fewer diagnostic tests and procedures, even after accounting for presenting features, while Medicare-insured patients–typically older with a higher comorbidity burden—more often undergo extensive evaluation, and uninsured or Medicaid-insured patients experience reduced diagnostic intensity. Our findings align with this broader literature and underscore the importance of examining how predictive systems may interact with existing disparities in care [30,39]. Future development should incorporate subgroup performance assessment and formal fairness analyses to mitigate the risk of reinforcing inequities [40,41].

Generalizability also warrants consideration. Although NHAMCS-ED provides a nationally representative picture of US emergency care, practice patterns, documentation structures, and diagnostic workflows can differ across individual hospitals, integrated health systems, and international ED settings. Variations in staffing models, triage protocols, and resource availability may influence both baseline testing rates and the predictors of ordering decisions. Accordingly, site-specific validation and prospective assessment are needed prior to operational deployment to ensure reliable performance across diverse clinical environments.

Finally, the clinical relevance of use prediction depends on its linkage to diagnostic quality and outcomes. While our models accurately predict the occurrence of laboratory testing, they do not evaluate appropriateness or downstream benefits (eg, diagnostic yield, treatment changes, ED length of stay, admission, and 72-h revisit). As such, this work should be viewed as a use-prediction study rather than an assessment of diagnostic quality. Prospective studies that pair deployment with adjudicated appropriateness criteria (eg, guideline-concordant ordering and choosing wisely targets) and patient-relevant outcomes are needed to establish clinical utility beyond use reduction.

Translating these models into decision support also requires attention to implementation details. Interpretability is essential for clinician trust; concise, case-level explanations (eg, SHAP-based summaries or simplified feature highlights) can clarify why a prediction was generated. User-interface design should minimize workflow disruption by surfacing predictions at natural decision points (eg, triage or during order entry) and offering actionable, context-aware cues rather than prescriptive directives. To avoid exacerbating alert fatigue, signals should be delivered judiciously, preferably through passive or noninterruptive displays with configurable thresholds and governance oversight. Addressing these considerations will be critical to realizing the potential of combined structured–unstructured models to support diagnostic stewardship in emergency care.

### Limitations

Despite the promising results, several limitations must be acknowledged. First, though nationally representative, the NHAMCS-ED dataset is cross-sectional, thus limiting our ability to further evaluate the long-term pattern of laboratory testing in future ED admissions, if any. Second, while the models performed well in predicting laboratory orders, they do not assess clinical appropriateness. Future work should aim to distinguish between necessary and unnecessary testing to directly address overuse. Third, laboratory test use was operationalized as a binary outcome, which does not capture the complexity of test selection, test combinations, or sequencing during ED encounters. While this approach supports scalable modeling and avoids arbitrary weighting of heterogeneous tests, more granular representations of laboratory ordering behavior may yield additional insights in future studies. Fourth, integrating predictive tools into clinical workflows requires tailored and setting-specific design, critical provider engagement, and diligent ethical oversight to ensure that the integration of ML models supports rather than replaces clinician judgment. Fifth, this study did not formally assess algorithmic fairness or evaluate subgroup performance using established fairness metrics (eg, disparate impact, equal opportunity, or calibration across race or ethnicity or insurance groups). Given the observed variation in laboratory testing across demographic and insurance strata, future research should incorporate formal fairness analyses to ensure that predictive models do not inadvertently reproduce or amplify existing inequities in diagnostic care. Sixth, this study did not assess the clinical appropriateness or downstream benefit of laboratory testing; as such, results reflect the prediction of test occurrence rather than diagnostic quality or impact on outcomes. Finally, the study could enhance its applicability by outlining practical steps for real-world implementation and validation within hospital environments. Specifically, it should address strategies for effectively integrating predictive insights into existing clinical workflows in a manner that is nonintrusive yet informative for clinicians, ensuring these models enhance rather than hinder decision-making processes.

### Conclusions

In summary, this study confirms the feasibility and added value of using combined structured and unstructured EHR data to predict laboratory test orders in ED settings. Such models can inform more intelligent diagnostic workflows by capturing quantifiable risk factors and nuanced clinical reasoning. Future work should focus on the real-time implementation and validation of diagnostic stewardship, aiming to reduce health care waste and improve patient outcomes in acute care environments.

## Supplementary material

10.2196/85255 Multimedia Appendix 1 Missing data pattern analysis.

## Abbreviations

AUC: area under the receiver operating characteristic curve
BERT: Bidirectional Encoder Representations from Transformers
ED: emergency department
EHR: electronic health record
ML: machine learning
NHAMCS-ED: National Hospital Ambulatory Medical Care Survey–Emergency Department
NLP: natural language processing
OR: odds ratio
ROC: receiver operating characteristic
SHAP: Shapley additive explanations
