# Supplementary material for: Predicting Laboratory Test Ordering in Emergency Departments Using Integrated Structured and Unstructured Electronic Health Records: Machine Learning Study
Source: JMIR Med Inform. 2026 Jun 15;14:e85255. doi: 10.2196/85255 (PMC13268631; doi:10.2196/85255)
Supplement: Multimedia Appendix 1 [file medinform-v14-e85255-s001.docx]

**Supplement 1 Missing Data Analysis**

| **`Variable** | **MissingCounts** | **MissingPercentage** |
| --- | --- | --- |
| **PAINSCALE** | 5023 | 0.38299656881433500 |
| **SEEN72** | 1264 | 0.09637819290888300 |
| **PAYTYPER** | 1242 | 0.09470072436141820 |
| **EPISODE** | 1148 | 0.08753335874952350 |
| **TEMPF** | 833 | 0.06351505909264200 |
| **POPCT** | 772 | 0.058863896301944300 |
| **PULSE** | 697 | 0.05314525352649640 |
| **RESPR** | 665 | 0.050705299275638600 |
| **INJPOISAD** | 651 | 0.0496378192908883 |
| **BPDIAS** | 560 | 0.04269919939001140 |
| **BPSYS** | 556 | 0.042394205108654200 |
| **ARREMS** | 354 | 0.026991993900114400 |
| **RESIDNCE** | 339 | 0.025848265345024800 |
| **ARRTIME2** | 318 | 0.02424704536789940 |

| **Variable** | **LabTestRate_Observed** | **LabTestRate_Missing** | **AbsoluteDifference** |
| --- | --- | --- | --- |
| **SEEN72** | 0.6004556577504010 | 0.5284810126582280 | 0.07197464509217290 |
| **PAYTYPER** | 0.5974901035963950 | 0.5555555555555560 | 0.041934548040839600 |
| **EPISODE** | 0.5896214590122840 | 0.6341463414634150 | 0.04452488245113080 |
| **TEMPF** | 0.6002279758996910 | 0.49459783913565400 | 0.10563013676403600 |
| **POPCT** | 0.6072267682087010 | 0.3743523316062180 | 0.23287443660248400 |
| **PULSE** | 0.6054920277017230 | 0.3802008608321380 | 0.22529116686958600 |
| **RESPR** | 0.6047389558232930 | 0.38345864661654100 | 0.22128030920675200 |

| **PAINSCALE_missing** | **1.0** | **2.0** |
| --- | --- | --- |
| **0** | 0.18255475376630000 | 0.8174452462337010 |
| **1** | 0.2575071986836690 | 0.7424928013163310 |

| **PAINSCALE_missing** | **1.0** | **2.0** | **3.0** | **4.0** | **5.0** |
| --- | --- | --- | --- | --- | --- |
| **0** | 0.2596572013302640 | 0.010488616014325900 | 0.02929137886927600 | 0.6794576618060890 | 0.02110514198004610 |
| **1** | 0.24149806284976300 | 0.012268618166164400 | 0.0284115368058545 | 0.6950064571674560 | 0.022815325010761900 |

The proportion of missing data for each variable is summarized in Supplementary Table S1. With the exception of pain score (38.3%), all structured variables exhibited low to moderate levels of missingness (<10%).

To assess the plausibility of a missing-at-random (MAR) assumption, we examined patterns of missingness in relation to laboratory test utilization and observable visit characteristics. Pain score missingness was not associated with laboratory testing rates (59.6% for observed vs. 59.0% for missing), and was more frequent among ambulance arrivals, consistent with higher-acuity encounters in which clinical priorities may precede formal pain documentation.

For other administrative and visit-level variables, differences in laboratory testing rates between missing and observed groups were modest. Larger differences were observed for vital sign variables (temperature, oxygen saturation, pulse, and respiratory rate), reflecting emergency department workflows in which vital signs are preferentially measured in higher-acuity encounters that are also more likely to undergo laboratory evaluation.

Taken together, these patterns suggest that missingness in structured variables is largely driven by observable clinical context and documentation practices rather than outcome-dependent mechanisms, supporting the use of median imputation under a missing-at-random assumption.
